# Role of ABO Blood Group in SARS-CoV-2 Infection in Households

**DOI:** 10.3389/fmicb.2022.857965

**Published:** 2022-05-06

**Authors:** Ales Janda, Corinna Engel, Jonathan Remppis, Sigrid Enkel, Andreas Peter, Sebastian Hörber, Tina Ganzenmueller, Sarah Schober, Christof Weinstock, Eva-Maria Jacobsen, Dorit Fabricius, Maria Zernickel, Thomas Stamminger, Andrea Dietz, Hans-Jürgen Groß, Sebastian F. N. Bode, Anneke D. M. Haddad, Roland Elling, Maximilian Stich, Burkhard Tönshoff, Philipp Henneke, Klaus-Michael Debatin, Axel R. Franz, Hanna Renk

**Affiliations:** ^1^Department of Pediatrics and Adolescent Medicine, Ulm University Medical Center, Ulm University, Ulm, Germany; ^2^Center for Pediatric Clinical Studies, University Children’s Hospital Tübingen, Tübingen, Germany; ^3^University Children’s Hospital Tübingen, Tübingen, Germany; ^4^Center for Clinical Transfusion Medicine Tübingen, Tübingen, Germany; ^5^Institute for Clinical Chemistry and Pathobiochemistry, University Hospital Tübingen, Tübingen, Germany; ^6^Institute for Medical Virology and Epidemiology of Viral Diseases, University Hospital Tübingen, Tübingen, Germany; ^7^Department of Transfusion Medicine, Ulm University, Ulm, Germany; ^8^Institute for Clinical Transfusion Medicine and Immunogenetics, Ulm, Germany; ^9^Red Cross Blood Service Baden-Württemberg-Hessen, Ulm, Germany; ^10^Institute of Virology, Ulm University Medical Center, Ulm, Germany; ^11^Institute of Clinical Chemistry, Ulm University Medical Center, Ulm, Germany; ^12^Center for Pediatrics and Adolescent Medicine, Medical Center Freiburg, Germany and Faculty of Medicine, University of Freiburg, Freiburg, Germany; ^13^Institute for Immunodeficiency, Medical Center Freiburg, Germany and Faculty of Medicine, University of Freiburg, Freiburg, Germany; ^14^Department of Pediatrics I, University Children’s Hospital Heidelberg, Heidelberg, Germany

**Keywords:** SARS-CoV-2, ABO blood group, children, COVID-19, household transmission

## Abstract

An association between certain ABO/Rh blood groups and susceptibility to SARS-CoV-2 infection has been proposed for adults, although this remains controversial. In children and adolescents, the relationship is unclear due to a lack of robust data. Here, we investigated the association of ABO/Rh blood groups and SARS-CoV-2 in a multi-center study comprising 163 households with 281 children and 355 adults and at least one SARS-CoV-2 seropositive individual as determined by three independent assays as a proxy for previous infection. In line with previous findings, we found a higher frequency of blood group A (+ 6%) and a lower frequency of blood group O (−6%) among the SARS-CoV-2 seropositive adults compared to the seronegative ones. This trend was not seen in children. In contrast, SARS-CoV-2 seropositive children had a significantly lower frequency of Rh-positive blood groups. ABO compatibility did not seem to play a role in SARS-CoV-2 transmission within the families. A correction for family clusters was performed and estimated fixed effects of the blood group on the risk of SARS-CoV-2 seropositivity and symptomatic infection were determined. Although we found a different distribution of blood groups in seropositive individuals compared to the reference population, the risk of SARS-CoV-2 seropositivity or symptomatic infection was not increased in children or in adults with blood group A or AB versus O or B. Increasing age was the only parameter positively correlating with the risk of SARS-CoV-2 infection. In conclusion, specific ABO/Rh blood groups and ABO compatibility appear not to predispose for SARS-CoV-2 susceptibility in children.

## Introduction

Susceptibility to severe acute respiratory syndrome coronavirus 2 (SARS-CoV-2) infection and severity of coronavirus disease 2019 (COVID-19) are heterogeneous across different populations. This heterogeneity has been attributed to various individual risk factors (Williamson et al., 2020; Goel et al., 2021; Ng et al., 2021). The ABO blood group is a suspected candidate because of its known role in other infectious diseases (Cooling, 2015). Early in the pandemic, a report from China showed an association of blood group A with increased risk of SARS-CoV-2 infection and lower risk for individuals with group O (Li et al., 2020). A first large-scale genome-wide study reported similar results with respect to COVID-19 severity (Ellinghaus et al., 2020). Others demonstrated a protective role of blood group O compared to A regarding disease susceptibility or severity (Golinelli et al., 2020; Wu et al., 2020). However, current results are conflicting and several high-quality studies did not support ABO-dependent intrinsic susceptibility to SARS-CoV-2 infection or COVID-19 severity (Boudin and Dutasta, 2020; Zhang et al., 2020; Zietz et al., 2020; Anderson et al., 2021). One of the possible explanations could be an over-recruitment of individuals with blood group O in blood banks due to their universal donor status. Thus, studies using blood donors as a reference group would find that the frequency of blood group O is lower among their COVID-19 cases than in the blood bank reference population (Dzik et al., 2020; Pendu et al., 2021).

Several underlying mechanisms have been proposed to explain the association between ABO blood group and SARS-CoV-2 infection: First, genetic histo-blood group polymorphisms can lead to variable viral attachment. Second, it has been hypothesized that the SARS-CoV-2 receptor-binding domain (RBD) may act as a lectin and preferentially bind to blood group A glycoprotein expressed on respiratory epithelial cells of blood group A individuals, favoring SARS-CoV-2 infection, though this has been recently contradicted *in vitro* (Wu et al., 2021; Boukhari et al., 2022). Third, the interaction of the SARS-CoV-1 and -2 spike protein with the cellular angiotensin-converting enzyme 2 (ACE2) receptor is inhibited by anti-A natural antibodies (Guillon et al., 2008; Le Pendu and Ruvoën-Clouet, 2020).

Last, it has been suggested that natural anti-A and anti-B could neutralize the virus when transmission occurs in an ABO-incompatible manner and this may reduce virus transmissibility by at least 60% (Ellis, 2021). Natural anti-A and anti-B antibodies present in ABO-incompatible virus recipients could recognize and bind to A and/or B epitopes expressed on the viral envelope glycoproteins, prevent the interaction of the virus and ACE2 receptor, and consequently prevent entry into lung epithelial cells (Goel et al., 2021). This mechanism is supported by the finding that COVID-19 patients had significantly lower anti-A and anti-B antibody levels compared to controls (Deleers et al., 2021). Additionally, Boukhari et al. (2022) demonstrated only recently that ABO incompatibility between spouses strongly decreased the risk of SARS-CoV-2 transmission.

Although we and others have demonstrated lower SARS-CoV-2 susceptibility in children and adolescents compared to adults (Spielberger et al., 2021; Stich et al., 2021; Tönshoff et al., 2021), pediatric data on the impact of ABO blood group on SARS-CoV-2 infection susceptibility or COVID-19 severity are scarce (Bari et al., 2021). Moreover, the association between ABO/Rh blood group and SARS-CoV-2 infection within households has not been evaluated so far.

In this large multi-center family cohort study, we examined the prevalence of ABO/Rh blood groups in SARS-CoV-2 seropositive and seronegative children and adults. We evaluated the contribution of ABO/Rh blood group to SARS-CoV-2 susceptibility and symptomatic infection. Additionally, we investigated a role of ABO compatibility between infected and exposed individuals on the SARS-CoV-2 transmissibility.

## Materials and Methods

### Study Design and Cohort

This cohort study on ABO blood group forms part of a non-interventional, national multi-center SARS-CoV-2 seroprevalence study and includes 668 adults and children living in 164 households with at least one confirmed SARS-CoV-2 index case (Supplementary Methods). It was initiated by the University Children’s Hospitals in Freiburg, Heidelberg, Tübingen, and Ulm and approved by the independent ethics committee of each center. Blood samples and data for this sub-study were collected at the study sites in Tübingen and Ulm in July and August 2020 and at a second visit in February and March 2021. Informed written consent was obtained from all participants or their legal guardians prior to enrollment. All authors had access to primary clinical trial data. The study is registered at the German Clinical Trials Register (DRKS), study ID 00021521 (German Clinical Trials Register, 2020), and was conducted according to the Declaration of Helsinki and the Strengthening the Reporting of Observational Studies in Epidemiology (STROBE) reporting guidelines.

### Data and Sample Collection

Participants completed a questionnaire documenting number of household members, age, gender, date of infection, and COVID-19-related symptoms. Distribution of blood groups was compared to the reference population (Wagner et al., 1995).

Blood samples were drawn during study visits. SARS-CoV-2 serology was performed using two assays directed against the viral spike protein and one directed against the nucleocapsid protein. In general, results were interpreted as positive if at least the Euroimmun IgG and the Roche Elecsys pan Ig assay were positive. In samples with discordant results or equivocal results in the Euroimmun IgG, decision about seropositivity or seronegativity were made by considering the result of the ADVIA Centaur XPT SARS-CoV-2 IgG assay (Supplementary Methods). For blood group typing ABO (forward and reverse) and Rhesus D, blood groups were typed using the ERYTRA Automated System blood group analyzer (Grifols S.A., Barcelona, Spain). Column agglutination cards [DG Gel AB0/Rh (CR)] and reagent cells (Reverse-Cyte 0.8%) from Grifols were used.

### Statistical Analysis

Chi-square tests were applied to test for difference in frequencies, and a generalized mixed model accounting for clustered data (cluster = family) was used to estimate fixed effects of the factors under consideration [blood group of index case (anti-A/no anti-A), symptomatic index case, yes/no, rhesus factor (positive/negative), age group of index (child/adult), age group of exposed family member (child/adult), blood group of exposed family member (anti-A/no anti-A), and rhesus factor of exposed family member (positive/negative)] on the risk of SARS-CoV-2 seropositivity and of age group, blood group, and rhesus factor on the occurrence of symptoms.

## Results

### General Characteristics of the Cohort

Blood group and SARS-CoV-2 antibodies were determined in 281 children and 355 adults. Mean age of children was 9 years (IQR 5;13), and that of adults was 43 years (IQR 38;50). Ten children and 98 adults were index cases. All other individuals were both exposed and infected (*n* = 244) or exposed and non-infected (*n* = 314). Eighty of 98 adult and 6 of 10 pediatric index cases showed symptoms including fever, cough, dysgeusia, and diarrhea (Table 1).

**TABLE 1:** Study cohort and patient characteristics.

	Children	Adults
All participants (%)	303 (45)	365 (55)
Number of individuals with blood sample taken (%)	281 (44)	355 (56)
Number of household members, mean ± SD	1.9 ± 0.74	2.23 ± 0.64
Mean age, years (25th; 75th centile)	9 (5;13)	43 (38;50)
Number of male patients (%)	153 (50)	180 (49)
Number of index cases (% of all index cases)	10 (7)	98 (93)
Number of exposed individuals (% of all exposed individuals)	293 (53)	264 (47)
Hospitalization (% of all participants within age-group)	0 (0)	4 (1)
SARS-CoV-2 seropositive (% within age-groups)	106 (38)	216 (61)
SARS-CoV-2 seronegative (% within age-groups)	175 (62)	139 (39)
Symptomatic index cases (% of index cases within age-groups)	6 (60)	80 (82)
Fever (% of index cases within age-groups)	6 (60)	65 (66)
Cough (% of index cases within age-groups)	3 (30)	58 (59)
Dysgeusia (% of index cases within age-groups)	4 (40)	55 (56)
Diarrhea (% of index cases within age-groups)	1 (10)	23 (23)

*Data are numbers and percentages as given in (). SD, standard deviation.*

### Frequency of ABO/Rh Blood Group in SARS-CoV-2 Seropositive and Seronegative Individuals

**FIGURE 1:**
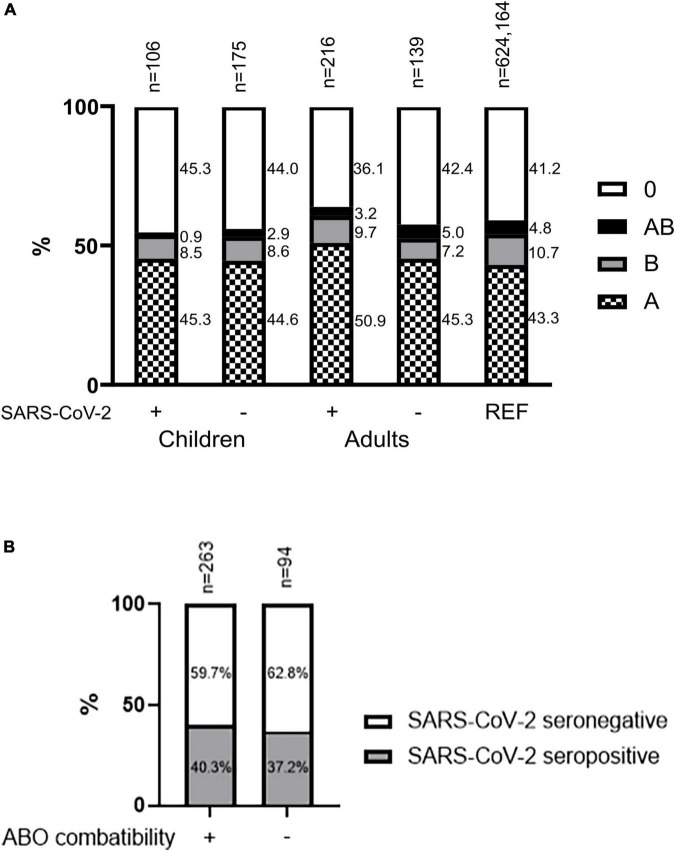
Overall observed blood group type distribution in children and adults **(A)** and frequency of SARS-CoV-2 transmission from index case to exposed individuals (*n* = 357) within the household cohort according to ABO blood group compatibility **(B)**. **(A)** Blood group prevalence in child and adult household members and in a German reference population (20). Numbers next to the columns represent the proportion of the particular blood group within the subject group (in percent). Rh, rhesus factor. **(B)** The black bar indicates the proportion of SARS-CoV-2 seropositive individuals, and the white bar indicates the proportion of SARS-CoV-2 seronegative individuals.

In our cohort, 38% of children and 61% of adults were SARS-CoV-2 seropositive. The overall distribution of blood group frequency did not differ by serostatus. Frequency of blood group A was 45% among SARS-CoV-2 seronegative and 49% among SARS-CoV-2 seropositive individuals, whereas frequency of blood group O was 39 and 43%, respectively (Figure 1 and Table 2A). As shown in Table 2A the overrepresentation of blood group A along with an underrepresentation of blood group O was only seen in adults, not in children. In contrast, Rh-positive blood groups were present more frequently among seronegative individuals (*p* = 0.02), and the effect can be mainly attributed to the difference found in children (77% seropositive versus 88% seronegative; *p* = 0.01) (Table 2A).

**TABLE 2A:** Blood type distribution within the cohort among SARS-CoV-2 seropositive and seronegative children and adults.

Blood group	Children (*n* = 281)			Adults (*n* = 355)			Total household cohort (*n* = 636)		
			
	SARS-CoV-2 seropositive (*n* = 106)	SARS-CoV-2 seronegative (*n* = 175)	Total	SARS-CoV-2 seropositive (*n* = 216)	SARS-CoV-2 seronegative (*n* = 139)	Total	SARS-CoV-2 seropositive (*n* = 322)	SARS-CoV-2 seronegative (*n* = 314)	Total
A	48 (45.3)	78 (44.6)	126 (44.8)	110 (50.9)	63 (45.3)	173 (48.7)	158 (49.1)	141 (44.9)	299 (47.0)
AB	1 (0.9)	5 (2.9)	6 (2.1)	7 (3.2)	7 (5.0)	14 (4.0)	8 (2.5)	12 (3.8)	20 (3.1)
B	9 (8.5)	15 (8.6)	24 (8.5)	21 (9.7)	10 (7.2)	31 (8.7)	30 (9.3)	25 (8.0)	55 (8.7)
O	48 (45.3)	77 (44.6)	125 (44.5)	78 (36.1)	59 (42.4)	137 (38.6)	126 (39.1)	136 (43.3)	262 (41.2)
*P*	0.76			0.43			0.47		
Rh-positive	82 (77.4)	154 (88.5)	236 (84.3)	178 (82.8)	120 (86.3)	298 (84.2)	260 (81.0)	274 (87.5)	534 (84.2)
*P*	0.01			0.37			0.02		

*One-way chi-square test for specified proportions. Data are numbers of participants and percentages given in brackets within the particular age-group and SARS-CoV-2 seropositive or seronegative group. p-value for comparison of blood group distribution and frequency of Rh rhesus factor between groups.*

In comparison to the reference population, the distribution of blood groups among all SARS-CoV-2 seropositive individuals was different (marginally significant; *p* = 0.05) (Table 2B). The frequency of blood group A was 6.1% higher and the frequency of blood group O was 1.9% lower compared to the reference population. This effect was not seen when comparing the SARS-CoV-2 seronegative individuals to the reference population (*p* = 0.24). In comparison to the reference population, Rh-positive blood groups were present more frequently in seronegative individuals (+ 4.5%; *p* = 0.03). Although as well present in adults (+ 3.3; *p* = 0.3), this effect can also be mainly attributed to the difference found in children (+ 5.5%; *p* = 0.05) (Table 2B).

**TABLE 2B:** Differences in the frequency of each blood group among SARS-CoV-2 seropositive and seronegative individuals compared to the reference population.

Blood group	Children (*n* = 281)		Adults (*n* = 355)		Total household cohort (*n* = 636)	
			
	SARS-CoV-2 seropositive (*n* = 106)	SARS-CoV-2 seronegative (*n* = 175)	SARS-CoV-2 seropositive (*n* = 216)	SARS-CoV-2 seronegative (*n* = 139)	SARS-CoV-2 seropositive (*n* = 322)	SARS-CoV-2 seronegative (*n* = 314)
A	+ 2.3	+1.6	+ 7.9	+2.3	+ 6.1	+1.9
AB	−4.1	−2.1	−1.8	± 0	−2.5	−1.2
B	−2.5	−2.4	−1.3	−3.8	−1.7	−3.0
0	+ 4.3	+3.0	−4.9	+ 1.5	−1.9	+ 2.3
*P*	0.2	0.39	0.11	0.56	0.05	0.24
Rh-positive	−6.0	+ 5.5	0	+3.3	−2	+ 4.5
*P*	0.12	0.05	0.93	0.30	0.34	0.03

*One-way chi-square test for specified proportions; percentage is shown, apart from p. p-value for comparison to the reference population in south-western Germany (26) with a frequency of blood group A of 43%; AB, 5%; B, 11%; O, 41%; Rh-positive, 83%; Rh-negative, 17%. Number of participants for Rh-factor are slightly different (children n = 280, adults n = 354, total = 634) due to missing values or atypical Rh antigens.*

*Rh, rhesus factor.*

### SARS-CoV-2 Seropositivity of Exposed Individuals According to ABO Compatibility

In total, 263 exposed individuals had an ABO-compatible blood group compared to the index case and 94 had an incompatible blood group. A total of 106 ABO-compatible and 35 ABO-incompatible transmissions occurred and the frequency of transmission was similar in both groups (40.3 and 37.2%; Figure 1B).

### Analysis of Risk Factors for SARS-CoV-2 Seropositivity and Symptomatic Disease

The cluster analysis did not reveal an increased odds ratio (OR) for SARS-CoV-2 seropositivity in individuals with blood group A or AB versus O or B, neither in children nor in adults. However, children with Rh-positive blood groups were twice as vulnerable to SARS-CoV-2 infection compared to those with Rh-negative blood groups [cluster-adjusted OR of 2.1 (95% CI 1.0–4.6)]. In the total cohort, compatibility of ABO blood group did not influence the risk for SARS-CoV-2 infection [cluster-adjusted OR of 1.2 (95% CI 0.6–2.3), Table 3].

**TABLE 3:** Risk of household members having blood group AB or A versus 0 or B or being Rh-negative versus Rh-positive for SARS-CoV-2 seropositivity.

Variable	Cluster-adjusted OR (95% CI)	*p*
**Children (*n* = 281)**		
Blood group AB or A versus 0 or B	0.97 (0.54-1.72)	0.91
Rh- versus Rh +	2.1 (1.0-4.6)	0.05
**Adults (*n* = 355)**		
Blood group A or AB versus 0 or B	1.18 (0.74-1.89)	0.47
Rh- vs Rh +	1.27 (0.67-2.41)	0.47
**Total cohort (*n* = 636)**		
Blood group A or AB versus 0 or B	1.18 (0.81-1.70)	0.39
Rh- vs Rh +	1.5 (0.9-2.5)	0.11
ABO compatible versus incompatible blood group between index case and exposed individuals	1.2 (0.6-2.3)	0.57

*Generalized linear mixed model. Odds ratio accounting for clustering of blood groups within households. ABO compatibility was only assessed for the total cohort, because only 6 index cases were children. p-value for cluster-adjusted OR. OR, odds ratio; CI, confidence interval; Rh, rhesus factor.*

Although the risk for SARS-CoV-2 infection in exposed individuals was slightly increased for adults relative to children, the ABO/Rh blood group was not associated with increased risk of SARS-CoV-2 infection (Table 4A). Additionally, ABO/Rh blood groups were not specifically associated with symptomatic infections. The only variable associated with the occurrence of symptoms in SARS-CoV-2 infection was an age above 18 years [OR 5.48 (95% CI 1.81–16.57)] (Table 4B).

**TABLE 4A:** Risk factors for SARS-CoV-2 seropositivity and symptomatic infection. Generalized linear mixed model for risk of SARS-CoV-2 seropositivity of exposed household members.

Variable	OR (95% CI)	*p*
**Index case**		
Adult versus child index case	19.6* (1.9–204.5)	0.01
Symptomatic index case	1.4 (0.5–3.4)	0.51
**Exposed individuals**		
Adult versus child exposed	1.8 (0.9–3.5)	0.08
Blood group of the exposed individual A or AB versus 0 or B	0.93 (0.44–2.0)	0.86
Rh-negative status versus Rh-positive status of the exposed individual	0.74 (0.28–2.0)	0.55

Generalized linear mixed model. p-value for cluster-adjusted OR. *Unreliable estimate, mostly due to the assumption of a parametric distribution, which is unstable for small numbers [here: 10 pediatric (9%) vs. 98 (91%) adult index cases].

*OR, odds ratio; CI, confidence interval; Rh, rhesus factor.*

**TABLE 4B:** Generalized linear mixed model for risk of symptomatic SARS-CoV-2 infection.

Variable	OR (95% CI)	*p*
Adult versus child	5.48 (1.81–16.57)	0.003
Blood group A or AB versus 0 or B	0.78 (0.30–1.99)	0.60
Rh-negative status versus Rh-positive status	1.65 (0.53–5.20)	0.38

*Generalized linear mixed model. p-value for cluster-adjusted OR. OR, odds ratio; CI, confidence interval; Rh, rhesus factor.*

## Discussion

This study found blood group A to be moderately overrepresented and blood group O to be moderately underrepresented in SARS-CoV-2 seropositive as compared to seronegative individuals and the reference population (Golinelli et al., 2020; Zietz et al., 2020; Ray et al., 2021). We observed this effect in adults, as previously reported by others (Pendu et al., 2021), but not in children. When considering the clustering of data in families, we found no evidence for blood groups A or AB as a substantial risk factor for SARS-CoV-2 infection in children or adults. This contrast to other studies might be explained by geographical, genetic, and methodological differences (Barnkob et al., 2020; Anderson et al., 2021). Here, we found a significantly lower frequency of Rh-positive blood groups among SARS-CoV-2 seropositive children and being Rh-negative doubled the risk for SARS-CoV-2 seropositivity. This contradicts previous reports in which Rh positivity constituted a risk factor for SARS-CoV-2 infection or severe COVID-19 (Latz et al., 2020; Zietz et al., 2020; Ray et al., 2021). Only one study from Sudan mentions a possible protective effect of presence of Rh-factor (Taha et al., 2020). A different ABO/Rh prevalence in the ethnically different Sudanese population prevents a comparison with our study. We can only hypothesize that the potential differences between adults and children may reflect a different underlying mechanism of ABO blood groups on SARS-CoV-2 infection.

Additionally, we investigated whether ABO incompatibility could potentially influence the risk for SARS-CoV-2 infection within the families. The frequency of transmission between index cases and exposed individuals was equally distributed between the ABO-compatible and ABO-incompatible group, and the risk for SARS-CoV-2 infection was not increased in ABO-compatible individuals in our cohort. This finding could be at least partially influenced by the natural phenomenon of a higher frequency of compatible blood groups within family cohorts due to inheritance of blood type, which makes ABO-compatible SARS-CoV-2 infections more likely. Therefore, to prove or reject the hypothesis of an effect of ABO compatibility on the risk of SARS-CoV-2 infection, transmission events need to be investigated in a cohort of unrelated individuals with known blood groups.

Since households represent the most important location for SARS-CoV-2 transmission, our data inform on the most common clinical scenario. In our cohort, the only parameters that positively correlated with the risk of intrafamilial transmission was the age of the exposed individual and the presence of symptoms in the index case, as described previously (Stich et al., 2021). Here, we confirm age group as a contributing factor, whereas ABO/Rh blood groups are not relevant for the risk of developing symptoms (Davies et al., 2020).

The strength of this multi-center study is its relatively large number of participants, and a separate analysis of infected and exposed children. Blood group determination within a household cohort of exposed SARS-CoV-2 seropositive and seronegative individuals allows controlling for genetic background and environmental influences. Nevertheless, the selection of participants and data collection may have been biased by voluntary participation and self-reporting of symptoms. Furthermore, we did not record data on ethnicity, and despite a relatively ethnic homogeneity in Southern Germany, this may have impacted the analysis. Finally, the findings apply to the SARS-CoV-2 variants circulating in Germany in spring 2020, and their validity in case of later virus variants (e.g., Delta and Omicron) is unclear.

In summary, we found that blood group A was moderately overrepresented in SARS-CoV-2-infected individuals, and the Rh-negative blood group was overrepresented in SARS-CoV-2-infected children. ABO compatibility did not increase the risk for SARS-CoV-2 infection. Individual ABO/Rh blood groups and ABO compatibility were not independent risk factors for SARS-CoV-2 acquisition, neither in children nor in adults.

## Data Availability Statement

The original contributions presented in the study are included in the article/Supplementary Material, further inquiries can be directed to the corresponding author/s.

## Ethics Statement

The studies involving human participants were reviewed and approved by Ethics Committees of the University Hospitals Tübingen, Ulm, Freiburg and Heidelberg. Written informed consent to participate in this study was provided by the participants’ legal guardian/next of kin.

## Author Contributions

AJ, HR, AF, and CE conceived the study and designed the experiments. AJ, HR, RE, AF, PH, BT, MS, and K-MD procured the funding. AP, SH, TS, AD, H-JG, and TG performed and interpreted the serological assays. SE and CW performed the blood grouping experiments. HR, AJ, AF, CE, DF, MZ, E-MJ, SFNB, and JR collected the samples or organized the collection. AJ, HR, AF, CE, and K-MD curated the data. CE, AJ, and HR performed the data analysis and generated the tables. AJ and HR wrote the first draft of the manuscript. SS, JR, TG, BT, MS, DF, SNFB, ADMH, and CE revised the first draft of the manuscript critically for important intellectual content. All authors approved the final version of the manuscript and confirm that they had full access to all the data in the study and accept responsibility for the decision to submit for publication.

## Conflict of Interest

The authors declare that the research was conducted in the absence of any commercial or financial relationships that could be construed as a potential conflict of interest.

## Publisher’s Note

All claims expressed in this article are solely those of the authors and do not necessarily represent those of their affiliated organizations, or those of the publisher, the editors and the reviewers. Any product that may be evaluated in this article, or claim that may be made by its manufacturer, is not guaranteed or endorsed by the publisher.
