# Supplementary material for: Role of ABO Blood Group in SARS-CoV-2 Infection in Households
Source: Front Microbiol. 2022 May 6;13:857965. doi: 10.3389/fmicb.2022.857965 (PMC9120758; doi:10.3389/fmicb.2022.857965)
Supplement: Supplementary file 1 [file Data_Sheet_1.docx]

- Supplementary Material -

## **Supplementary Methods**

## Study conditions

Blood samples for SARS-CoV-2 serology were drawn 8-12 weeks after the first pandemic wave in Germany in the Spring 2020, when the incidence had fallen from about 35-50/100.000 to about 8/100.000 in southern Germany ^1, 2^. Blood group was determined either in the first set of blood samples or in a follow-up sample taken 11-12 months later.

## Study cohort


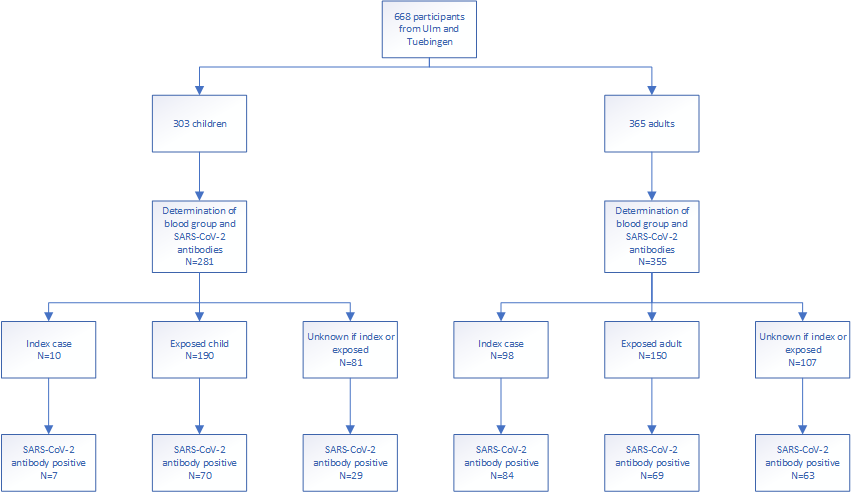


## Recruitment and eligibility

Households meeting the eligibility criteria were recruited via the local health authorities. Inclusion criteria were met if (1) SARS-CoV-2 had been previously detected by RT-PCR from a nasopharyngeal or oropharyngeal swab in at least one household member as reported by the local health authority (2) at least one household member was under the age of 18 years, (2) participants resided in the state of Baden-Württemberg, (4) all household members had been officially released from quarantine. Participants were excluded if written informed consent was missing or they had insufficient knowledge of the German language to understand the participant information forms.

## Index case definition

The index case was defined as the participant within the household with the earliest PCR-proven SARS-CoV-2 infection (reported by local health authorities); index cases had to additionally be seropositive within this study and either be the first person within their household to have symptoms or be the (only) person in the household with close contact to a SARS-CoV-2 case prior to infection. If two family members were tested positive <48h apart or if secondary cases occurred >14 days apart, the family was excluded. However, we additionally performed the analysis in both datasets (also including the families with more than 1 possible index case) and did not find any differences. All other household members were defined as “exposed individuals” and classified as seropositive or -negative for SARS-CoV-2 antibodies reflecting SARS-CoV-2 infection. For interpretation of serology results, please see Supplementary Table 2.

## SARS-CoV-2 Serology

### Serum samples were obtained and stored at -20°C until processed further.

## SARS-CoV-2 IgG ELISA

The 96-well SARS-CoV-2 IgG ELISA assay (EI 2606-9601 G Euroimmun AG, Lübeck, Germany) was performed on an automated BEP 2000 Advance system (Siemens Healthcare Diagnostics) according to the manufacturer’s instructions. The ELISA assay detects anti-SARS-CoV-2 IgG directed against the S1 domain of the viral spike protein and relies on an assay-specific calibrator to report a ratio of specimen absorbance to calibrator absorbance. The final interpretation of positivity is determined by ratio above a threshold value given by the manufacturer: positive (ratio ≥1.1), borderline (ratio 0.8–1.0) or negative (ratio <0.8). Quality control was performed following the manufacturer’s instructions on each day of testing.

## Elecsys anti-SARS-CoV-2 immunoassay

The Elecsys anti-SARS-CoV-2 assay is an electrogenerated chemiluminescence immunoassay (Roche Diagnostics) and was used according to manufacturer’s instructions (v.1.0, as constituted in May 2020). It is intended for the detection of high-affinity antibodies (including IgG) directed against the nucleocapsid protein of SARS-CoV-2 in human serum. Readout was performed on a Cobas e411 analyzer. Negative results were defined by a cutoff index of <1.0. Quality control was performed following the manufacturer’s instructions on each day of testing.

## ADVIA Centaur XPT SARS-CoV-2 IgG (COV2G) Assay

The ADVIA Centaur XPT SARS-CoV-2 IgG (COV2G) assay is a fully automated chemiluminescent immunoassay (CLIA) for high throughput random access analyzers. Samples were processed according to the manufacturer’s procedures with the specified controls and calibrators on the Advia Centaur XP platform (Siemens, Munich, Germany). The assay is intended for qualitative and semi-quantitative detection of IgG antibodies to the S1 receptor-binding domain (RBD) antigen of SARS-CoV-2 in human serum.

Sera with concordant results in one assay directed against the viral N protein (Elecsys® Anti-SARS-CoV-2 IgG/IgM ECLIA) and the Euroimmun Anti-SARS-CoV-2-ELISA (IgG) directed against the viral S protein were categorized as seropositive or seronegative. In the case of discordant or equivocal results, the semi-quantitative Siemens sCOV2G (CLIA), directed against the viral S1 RBD, was performed. Sera with a positive reaction in this additional assay were classified as seropositive. + positive; - negative; +/- equivocal. Samples with discordant findings were reviewed independently by two laboratory physicians to verify the final result according to the interpretation matrix.

## Serology Interpretation Matrix

| **SARS-CoV-2 pan Ig Roche** | **CoV IgG**  **Euroimmun** | **sCOVG IgG**  **Siemens** | **Final result** |
| --- | --- | --- | --- |
| + | + | + | positive |
| -- | - | - | negative |
| + | + | - | positive |
| - | - | + | negative |
| + | - | + | positive |
| + | - | - | negative |
| - | + | + | positive |
| - | + | - | negative |
| + | +/- | + | positive |
| + | +/- | - | negative |
| - | +/- | + | positive |
| - | +/- | - | negative |

## **3. Supplementary References**

1. Federal State of Baden-Wuerttemberg Lha. Health atlas of Baden-Wuerttemberg. Accessed 09.12.2021, 2021. <http://www.gesundheitsatlas-bw.de/dataviews/report?reportId=64&viewId=212&geoReportId=381&geoId=3&geoSubsetId>=

2. Institute RK. Daily situation report of the RKI concerning COVID-19. Accessed 09.12.2021, 2021. <https://www.rki.de/DE/Content/InfAZ/N/Neuartiges_Coronavirus/Situationsberichte/2020-03-21-de.pdf?__blob=publicationFile>
